# Procyanidin trimer C1 reactivates latent HIV as a triple combination therapy with kansui and JQ1

**DOI:** 10.1371/journal.pone.0208055

**Published:** 2018-11-26

**Authors:** Daniele C. Cary, B. Matija Peterlin

**Affiliations:** Departments of Medicine, Microbiology and Immunology, University of California at San Francisco, San Francisco, California, United States of America; Baylor College of Medicine, UNITED STATES

## Abstract

Although anti-retroviral therapies have greatly extended the lives of HIV infected individuals, current treatments are unable to completely eliminate virally infected cells. A number of latency reversing agents have been proposed for use in a “shock and kill” strategy to reactivate latent HIV, thus making it vulnerable to killing mechanisms. Procyanidin trimer C1 (PC1) is a flavonoid found in multiple plant sources including grape, apple, and cacao, which has antioxidant and anti-inflammatory properties. We determined that PC1 reactivates latent HIV in cell line and primary cell models of HIV, through activation of the MAPK pathway. Notably, PC1 reactivates latent HIV without increasing surface markers of T cell activation. Combining several therapeutics, which activate HIV transcription through different mechanisms, is the most efficient approach to clinically reactivate latent reservoirs. We utilized PC1 (MAPK agonist), kansui (PKC agonist), and JQ1 (BET bromodomain inhibitor) in a triple combination approach to reactivate latent HIV in cell line and primary cell models of HIV latency. When used in combination, low concentrations which fail to reactivate HIV as single treatments, are effective. Thus, several mechanisms, using distinct activation pathways, act together to reactivate latent HIV.

## Introduction

Latency is the principal obstacle to complete eradication of HIV [1]. Transcriptionally silent virus evades anti-retroviral therapies (ART), which target viral proteins expressed during the replication cycle [2, 3]. In spite of undetectable plasma viremia while on ART, suppressed patients have rapid viral rebound following treatment interruption [4, 5]. As a consequence, HIV+ individuals must remain on life-long ART. Although HIV infection in the era of ART has become a more manageable chronic infection, problems with adherence to drug regimens, co-morbidities, and the emergence of drug resistance emphasize the need for continued research into HIV cure [6–8]. Since the barrier to cure is the persistence of latent HIV, targeting this persistent and transcriptionally silent virus is critical.

A “shock and kill” strategy has been proposed to target latently infected cells, in which virus is shocked out of latency by compounds which activate HIV transcription, making virally infected cells available to cellular and immune killing mechanisms [9]. HIV is dependent on cellular transcriptional machinery, including nuclear factor kappa B (NF-κB), activator protein 1 (AP1), and positive transcription elongation factor b (P-TEFb) [10]. P-TEFb is a critical transcription factor (TF) required for HIV gene expression. Cellular P-TEFb exists in equilibrium between an inactive state, bound to Hexim1 and 7SK snRNA, and a free active state that is recruited to the HIV long terminal repeat (LTR) by NF-κB and the HIV transactivator of transcription (Tat) [11, 12]. Once at the LTR, P-TEFb phosphorylates negative elongation factors and RNA polymerase II (RNAPII), thus permitting productive elongation [13]. NF-κB is regulated by its inhibitor, IκBα [14]. Upon activation, IκBα is phophorylated, degraded, and released; freeing NF-κB for nuclear transclocation. The mitogen activated protein kinases (MAPK) signaling cascade phosphorylates JNK, p38, and extracellular signal-related kinase (ERK), which regulate AP1 expression [15]. Proposed latency reversing agents (LRAs) target these TF to reactivate latent HIV and purge hidden HIV reservoirs.

A number of LRAs are currently being investigated to determine which reactivate latent HIV through separate but complementary cellular pathways; and each group of LRA has its own advantages and caveats [16]. Histone deacetylase and BET bromodomain inhibitors (HDACi and BETi), such as JQ1, robustly activate HIV through the release of P-TEFb from 7SK snRNP [17, 18]. However, these compounds do not work in human primary T cells [19, 20]. Resting CD4+ T cells have low expression of P-TEFb and other cyclin dependent kinases (CDKs) which are abundant in T cell lines and necessary for efficient transcriptional elongation and co-transcriptional processing of nascent transcripts [10, 21]. Therefore, it is crucial to include a compound that increases P-TEFb levels for JQ1 to function properly. Protein kinase C (PKC) agonists, including ingenol, induce a robust T cell response including activation of inflammatory response genes and cell proliferation [22–25]. A crude extract from Euphorbia kansui (kansui) contains 12 ingenols and other bioactive compounds, which may contribute to its overall ability to activate T cells without much toxicity [26–28]. Kansui reactivates latent HIV in cell lines and primary T cell models of latency, increases cellular levels of the P-TEFb components cyclin T1 (CycT1) and CDK9, and reactivates latent virus from cells isolated from ART suppressed patients [21]. In spite of the appeal of PKC agonists, there has been caution using these agents as a single treatment since concentrations which reactivate latent HIV could induce global T cell activation and cytokine release if delivered clinically [29, 30]. Ideally, a lower dose of PKC agonist could be used in combination with other compounds which reactivate HIV, but do not activate T cells. MAPK signaling activates HIV through induction of AP1 expression [31, 32]. Procyanidin C1 trimer (PC1), a flavonoid isolated from Theobroma cacao (cacao), reactivates cell line models of HIV through the MAPK pathway and act synergistically with the potent PKC agonist PMA [33]. PC1 is also commercially available as a general wellness supplement, derived from a variety of plant sources including grape seed. The use of MAPK agonists to reactivate latent HIV has not been investigated as rigorously as PKC, HDACi, or BETi, but has great potential for use in combinatorial therapeutic approaches. The most effective proposed latency reversal strategies include two or more compounds which reactivate latent HIV by different mechanisms [21, 29, 30]. Thus, several mechanisms, using distinct activation pathways, will act together to reactivate latent HIV.

In this study, we determined the potential of PC1 isolated from grape seed as an LRA. Using cell line and primary CD4+ T cell models of HIV latency, we found that PC1 reactivates latent HIV without concurrently activating these lymphocytes. Indeed, the combination of PC1, kansui, and JQ1 activated cellular TFs via separate pathways and reactivated HIV synergistically from latency.

## Materials and methods

### Cell lines, PBMC, and primary CD4+ T cells

2D10 cells (obtained from Dr. Jonathan Karn at Case Western Reserve University) are a Jurkat based HIV latency cell line model that contains attenuated Tat and d2EGFP in the place of Nef. Reactivation of latent HIV in 2D10 cells was measured by d2EGFP expression by flow cytometry. Trima residuals from healthy donors, from Trima aphoresis collection and enriched for PBMC, were obtained from Blood Center of the Pacific (San Francisco, CA). Bulk peripheral blood mononuclear cells (PBMCs) were cultured 3 days on tissue cultured plastic to allow for macrophages to adhere. Non-adherent PBMCs were negatively selected for purified CD4+ T cells, or used directly in PMBC experiments. CD4+ T cells were selected from bulk PBMC using negative bead selection (Dynal CD4+ untouched beads, Invitrogen). Primary CD4+ T cells were activated and expanded using CD3/CD28 beads (Invitrogen) and 30 U/ml interleukin 2 (IL-2) for 5 days.

### Procyandin Trimer C1

Procyanidin Trimer C1 (PC1) was obtained from ChemFaces Biochemical (CFN99560, Wuhan, China) and reconstituted in DMSO. Working stocks were stored at -20°C.

### Cell culture and reactivation conditions

Cells were maintained in RPMI 1640 supplemented with 10% FBS and Penicillin/Streptomycin at 37°C with 5% CO_2_. Cells were stimulated at a concentration of 1 x 10^6^ cells/ml with PC1 at indicated concentrations, DMSO (1 μl/ml), kansui extract (Baoji F.S. Biological Development Co. Ltd. (Shanxi, China)), or JQ1 (Martin Delaney Collaboratory of AIDS Researchers for Eradication (CARE)). Cells were seeded in triplicate wells of a 24 well plate, and stimulated for 24 hours. Approximately 5–10 x 10^6^ cells/5-10 ml on 10 cm plates were stimulated to obtain lysates for protein expression analysis.

### Generation of HIV-1 infectious titers and infections

Infectious stocks of HIV-1 were generated by transfecting 293T cells with 15 μg of pNL4.3-Nef(+)-HSA (NL4.3 HSA) (National Institutes of Health AIDS Research and Reference Reagent Program, Division of AIDS, NIAID, NIH: pNL4-3.HSA.R+.E- from Dr. Nathaniel Landau at the NYU School of Medicine [34, 35]) and 3 μg of VSV-G using calcium phosphate. Viral supernatants were harvested and filtered through a 0.45 μm disc at 48 hours post transfection. Approximately 0.5 x 10^6^ pg p24 of infectious virus were added per 1 x 10^6^ activated CD4+ T cells. Cells were spinoculated for 90 min at 2000 rpm with polybrene (2 μg/ml). 24 hours post infection, cells were washed twice to thoroughly remove initial infectious virus.

### Human CD4+ T cells model of HIV latency

Primary CD4+ T cells were activated, infected with HIV-1 NL4.3 HSA, and given decreasing concentrations of IL-2 over 12 days in culture to induce quiescence and HIV latency, as previously described [21]. Latently infected and uninfected control cells were stimulated for 24 hours and samples were collected for flow cytometry analysis.

### Flow cytometry analysis

Cells were harvested 24 hours post reactivation and washed in cold PBS and 0.5 x 10^6^ cells were allotted to each tube. Cells were stained with PE mouse anti-human CD69 (555531, BD Biosciences), PE anti-human CD25 (555432, BD Biosciences), FITC rat anti-mouse HSA (553261, BD Biosciences), or fixed immediately to analyze GFP expression. Cells were fixed in 2% paraformaldehyde and analyzed using the BD Biosciences FACScaliber and CellQuest Pro software at the UCSF Parnassus Flow Cytometry Core. Cells were gated on the live lymphocyte gate using the forward and side scatter plot, and the percentage of live lymphocytes in 10,000 collected total cells was used as an estimate for cell viability.

### Western blotting analysis of protein expression

Whole cell lysates were generated using lamemmli buffer (Bio-rad) in the presence of proteinase inhibitor cocktail. Lysates were run on 10% SDS-PAGE and transferred onto a nitrocellulose membrane. Membranes were cut at approximately 60 kDa; the top portion of the membrane was used to probe for CycT1 (75 kDa) and the bottom portion was used to probe for phospho-(p-) (42 kDa) and total IKBα (40 kDa), p- and total ERK (42 kDa), and β-actin (55 kDa). Membranes were blocked in 5% non-fat milk (NFM) for at least 1 hour and blotted overnight with mouse anti-human cyclin T1 antibody (sc-271348, Santa Cruz Biotechnology), mouse anti-human p-ERK (P-p44/42 MAPK) (9106S, Cell Signaling), rabbit anti-human ERK (p44/42 MAPK) (9102S, Cell Signaling), mouse anti-human p-IKBα (9246S, Cell Signaling), mouse anti-human IKBα (48145S, Cell Signaling) and rabbit anti-human β-actin (ab8227, abcam) antibodies in 5% NFM. Membranes were washed 3 x with PBS with 0.05% Tween 20, and then blotted for 1 hour with HRP anti-rabbit IgG, in 5% NFM. After washing 3 x with PBS with 0.05% Tween 20; membranes were treated with ECL Plus chemiluminescence reagent (Promega) for 5 minutes and imaged using Odyssey Fc imaging system and Image Studio software (LI-COR). Reprobed membranes were stripped with NewBlot Stripping Buffer (LI-COR) then washed 3 x with PBS.

### Calculation of drug synergy

To determine whether combination treatment resulted in synergistic activation of HIV we used the Bliss independence model previously described [29]. The equation fa_xy,p_ = fa_x_ + fa_y_- (fa_x_)(fa_y_), wherein fa_x,p_ is the prediction based on the individual effects of PC1 (fa_x_) and kansui + JQ1 (fa_y_). The individual effects were calculated by the equation fa_x/y/xy_ = (% GFP positive combination treatment—% GFP positive DMSO)/ (% GFP positive kansui 500 μg/ml—% GFP positive DMSO). The difference between the observed and predicted values (Δfa_xy_ = fa_xy,o_—fa_xy,p_) was calculated. A value greater than 0 signified synergy, a value equal to 0 indicated Bliss independence, and a value less than 0 indicated antagonism.

### Statistical analysis

Statistical analysis was performed using a Student *t* test, two-tailed distribution, and assuming equal variances. Further regression analysis was performed to confirm the significance of the triple combination treatments in relation to the effects of each single and double combination treatment, wherein an F statistic of less than 0.05 was considered significant.

## Results

### PC1 reactivates latent HIV in a cell line model of HIV latency

**Fig 1:**
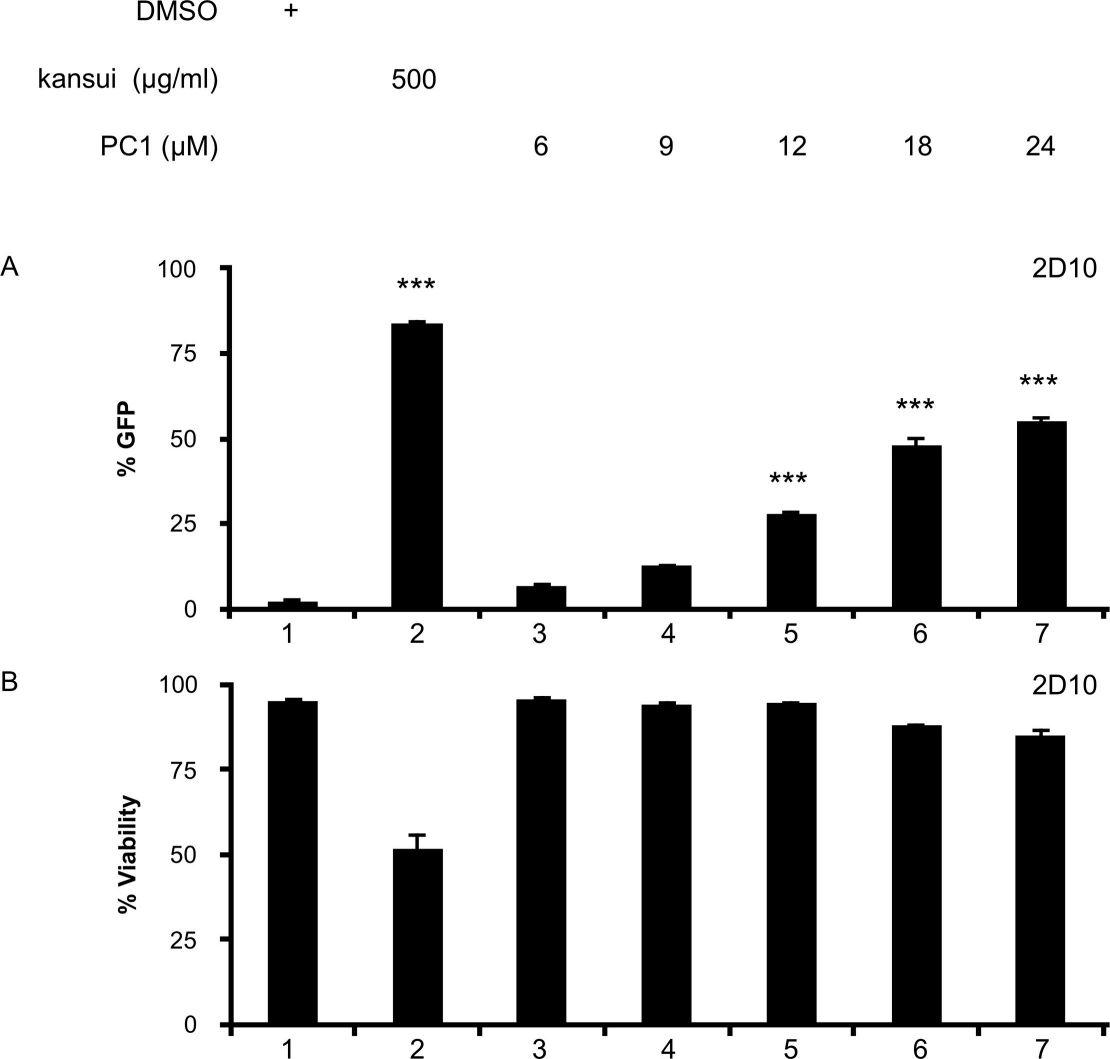
PC1 reactivates latent HIV in a cell line model of HIV latency. 2D10 cells were stimulated for 24 hours with DMSO, kansui, or PC1 at the indicated concentrations. (A) GFP expression was measured by flow cytometry. (B) Percent viability was estimated using forward/side scatter and the percentage of live lymphocytes in 10,000 total cells analyzed. Triplicate stimulations were performed. Error bars represent standard error of the mean. (***p<0.001).

For this study, we obtained PC1 isolated from grape seed, which is the source of many commercially available PC1 wellness supplements. Preliminary experiments were performed in 2D10 cells, which are Jurkat cells that stably express GFP driven by the HIV LTR [36]. GFP was measured by flow cytometry to determine HIV reactivation. A crude extract of kansui, containing the PKC agonist ingenol, was used as a positive control [21]. Kansui treatment resulted in a 42-fold increase in GFP expression over the DMSO control (Fig 1A, lane 2). A dose dependent increase in GFP was observed with increasing concentrations of PC1 (Fig 1A, lanes 3–7). GFP expression was significantly increased in cells treated with 12, 18, and 24 μM PC1, ranging from 12 to 24-fold over the DMSO control (Fig 1A, lanes 5–7). Importantly, none of the concentrations of PC1 tested were toxic to 2D10 cells (Fig 1B, lane 3–7) compared to kansui, which decreased their viability by 45% (Fig 1B, lane 2). These preliminary studies provided the proof of concept that PC1 from grape seed reactivates latent HIV, and effective concentrations were similar to those observed with PC1 from cacao [33].

### PC1 activates the MAPK pathway

**Fig 2:**
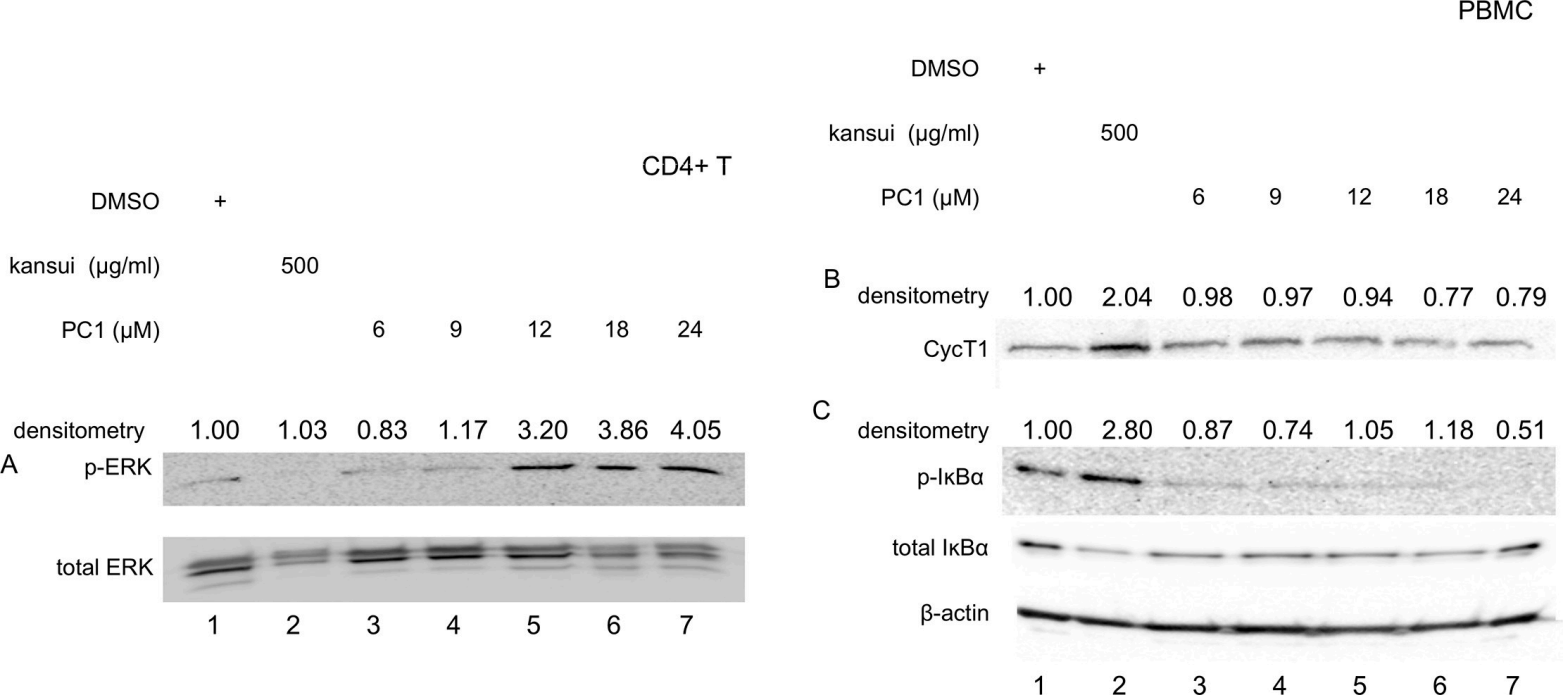
PC1 activates ERK. Human CD4+ T cell or PBMC were stimulated for 24 hours with DMSO, kansui, or PC1 at indicated concentrations. Whole cell lystates were separated on 10% SDS PAGE. Membranes were probed with specific antibodies for (A) p-ERK and total ERK (B) CycT1, (C) and p-IκBα and total IκBα, and β-actin. Densitometric data were collected, normalized to appropriate loading controls with the DMSO negative control set to one. Results are representative of western blots from three healthy donors.

PC1 from cacao activates the MAPK pathway, but not the PKC pathway, in a Jurkat model of HIV latency [33]. To determine if PC1 from grape seed activates the same cellular pathway, human primary CD4+ T cells were stimulated with increasing concentrations of PC1 for 24 hours. PC1 activated the MAPK signaling cascade through phosphorylation of ERK (p-ERK), where levels of total ERK protein remained constant (Fig 2A). A threshold of p-ERK was achieved in cells treated with 12 μM PC1 (Fig 2A, lane 5), and levels of phosphorylation did not increase with 18 and 24 μM PC1 (Fig 2A, lanes 6 and 7). In contrast, kansui did not induce p-ERK (Fig 2A, lane 2). These results agree with previously published reports that PC1 from cacao activates the MAPK signaling cascade [33].

PKC signaling activates NF-κB and increases cellular expression of P-TEFb [37]. Resting primary PBMC express low levels of CycT1, with some variability in expression between donors (Fig 2B, lane 1). Treatment with kansui induced a robust increase in CycT1 expression (Fig 2B, lane 2) and phophorylation of IκBα, as a measure of NF-κB activation (Fig 2C, lane 2). However, no concentration of PC1 tested increased levels of CycT1 or p-IκBα (Fig 2B and 2C, lanes 3–7).

### PC1 does not increase markers of T cell activation

**Fig 3:**
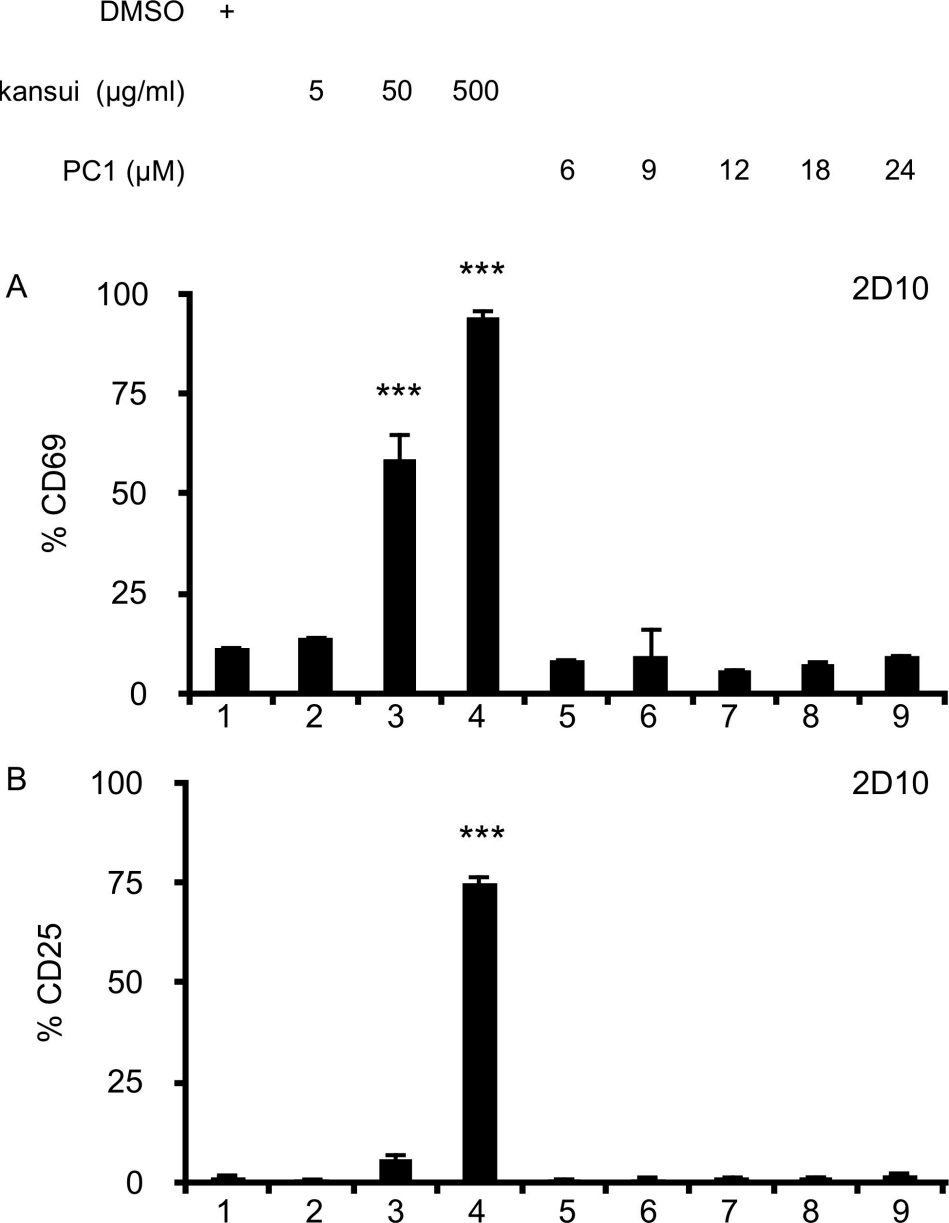
PC1 does not increase markers of T cell activation. 2D10 cells were stimulated for 24 hours with DMSO, kansui, or PC1 at the indicated concentrations. (A) CD69 and (B) CD25 expressions were measured by flow cytometric analyses. Triplicate stimulations were performed. Error bars represent standard error of the mean. (***p<0.001).

Treatment of 2D10 cells with kansui resulted in a dose dependent increase in the early T cell activation marker, CD69 (Fig 3A, lane 2–4). Kansui also induced expression of the high affinity IL-2 receptor, CD25. However, CD25 induction was more all or none, with only the highest dose of kansui inducing CD25 (Fig 3B, lanes 2–4). No concentration of PC1 tested increased CD69 or CD25 expression on 2D10 cells (Fig 3A and 3B, lanes 5–9). However, this finding is not surprising given the lack of NF-κB activation (Fig 2B, lanes 3–7).

### PC1 reactivates latent HIV in a primary CD4+ T cell model of HIV latency

**Fig 4:**
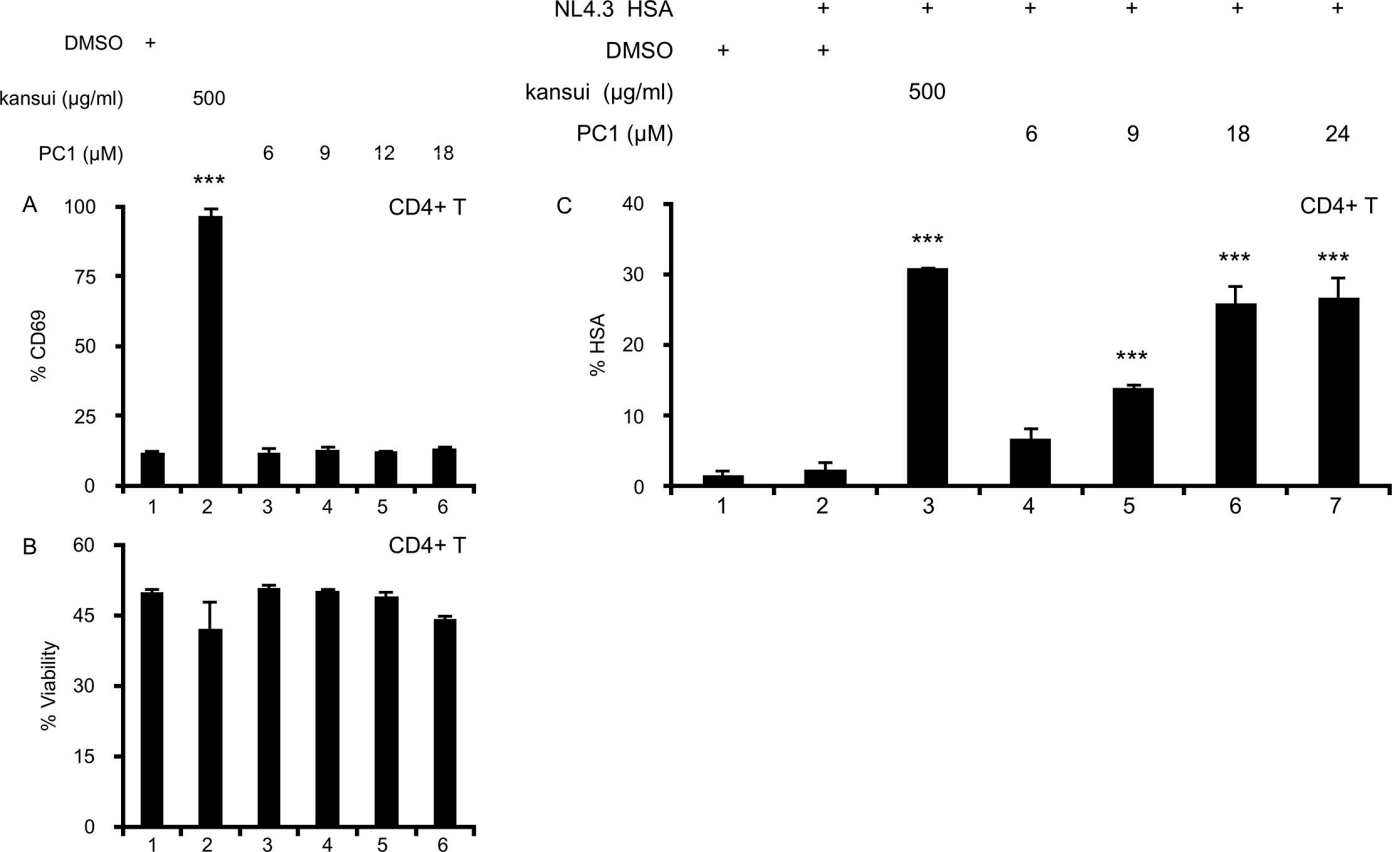
PC1 reactivates latent HIV in a primary CD4+ T cell model of HIV latency. (A) Uninfected human primary CD4+ T cells were stimulated for 24 hours with DMSO, kansui, or PC1 at the indicated concentrations. Cells were stained with anti-CD69 antibody and measured by flow cytometric analyses. (B) Percent viability was estimated using forward/side scatter and the percentage of live lymphocytes was determined in 10,000 total cells analyzed. (C) Human primary CD4+ T cells were infected with VSV-G-pseudotyped NL4.3 HSA and maintained over 12 days with decreasing concentrations of IL-2 to establish latency as previously described [21]. Uninfected cells were maintained in the same conditions. At 12 days post infection, cells were stimulated for 24 hours with DMSO, kansui, and PC1. Cells were stained with anti-HSA antibody. Data are presented as a fold change in HSA expression over the uninfected control cells. Experiments were repeated with three independent donors, each with three technical repeats. A representative experiment, from a single donor is presented. Error bars represent standard error of the mean. (***p<0.001).

While PC1 activated HIV transcription in 2D10 cells (Fig 1A), it was important to validate effects of proposed therapeutic reagents on primary cells. Immortalized cell lines express high levels of P-TEFb, CDK11, and NF-κB, which are lacking in resting CD4+ T cells [18, 38]. Therefore, it is important to verify that LRAs are not just effective in cell lines. In these cells, kansui stimulation resulted in robust CD69 expression (Fig 4A, lane 2), PC1 did not induce the expression of CD69 at any concentration tested (Fig 4A, lanes 3–6). Moreover, no concentration of PC1 reduced the viability of primary CD4+ T cells (Fig 4B, lanes 3–6).

**Fig 5:**
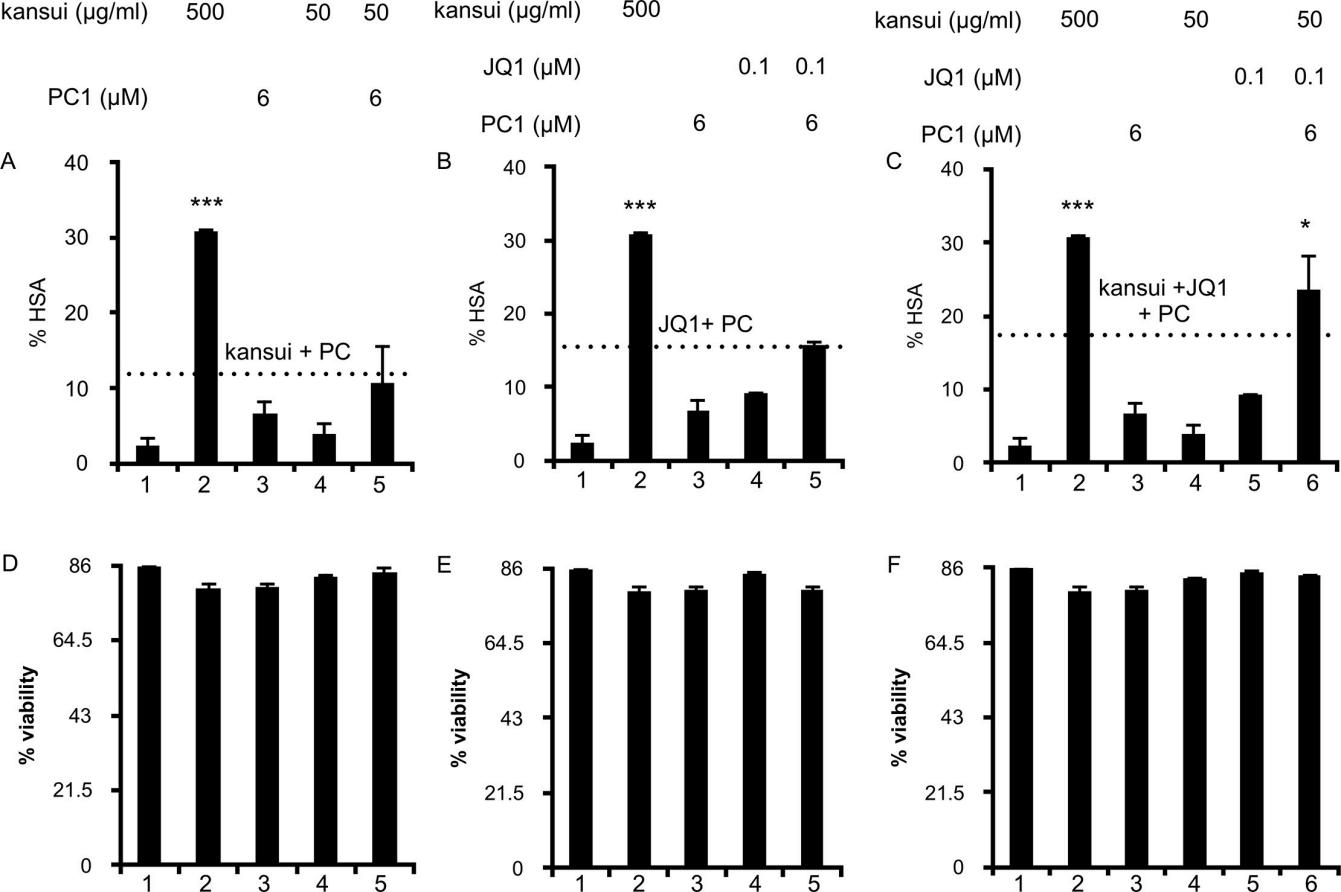
Combination therapy of low dose PC1, kansui, and JQ1 reactivates latent HIV in a primary CD4+ T cell model of HIV latency. Human primary CD4+ T cells were infected with VSV-G-pseudotyped HIV-1 NL4.3 HSA and maintained over 12 days with decreasing concentrations of IL-2 to establish latency as previously described. Uninfected cells were maintained in the same conditions. At 12 days post infection, cells were stimulated for 24 hours with: (A) single and double PC1 (6 μM) and kansui (50 μg/ml) (B) single and double PC1 (6 μM) and JQ1 (0.1 μM) (C) single and triple PC1 (6 μM), kansui (50 μg/ml), and JQ1 (0.1 μM). Cells were stained with anti-HSA antibody and measured by flow cytometric analyses. Data are presented as a fold change in HSA expression over uninfected control cells. Dashed line is equal to the additive effects (sum % HSA of the single treatments), and a synergistic effect was observed if double and triple treatments surpassed this line. Synergy was validated using Bliss Independence calculation of drug synergy. Experiments were repeated with three independent donors, each with three technical repeats. A representative experiment, from a single donor is presented. (D-F) Uninfected CD4 + T cells were treated as above. Percent viability was estimated using forward/side scatter and the percentage of live lymphocytes in 10,000 total cells analyzed. Error bars represent standard error of the mean. (*p<0.05, **p<0.01, ***p<0.001).

Using our *in vitro* HIV latency model [21], we tested the ability of PC1 to reactivate latent HIV in primary human CD4+ T cells. Activated T cells were infected with VSV-G-pseudotyped HIV-1 NL4.3 HSA R+E- (NL4.3 HSA) that expresses the murine heat shock antigen (HSA), which allows reactivated cells to be quantified using flow cytometry. Following infection, cells were driven into quiescence through gradually decreasing concentrations of IL-2, and at day 12 post infection, HSA was no longer detectable on the surface of infected cells (Fig 4C, lane 2). Uninfected cells were maintained in the same conditions (Fig 4C, lane 1). Cells were stimulated for 24 hours with kansui and indicated concentrations of PC1. Kansui induced a 15-fold increase in HSA expression (Fig 4C, lane 3). Reactivation in primary lymphocytes was approximately 2.5 fold less than observed in 2D10 (Fig 1 compared to Fig 5). This result was expected due to the abundant expression of key cellular transcription factors, such as P-TEFb, CDK11, and NF-κB in immortalized cell lines [18, 38], which make them easier to reactivate. This difference highlights the importance of verifying cell line responses in primary cell models. While 6 μM PC1 had no effect (Fig 4C, lane 4), 9 μM PC1 increased HSA expression 6-fold (Fig 4C, lane 5). At higher concentrations of PC1, 18 and 24 μM, a 12.5-fold increase was observed, nearly equivalent to that of the kansui positive control (Fig 4C, lane 6 and 7). Thus PC1 alone also reactivates latent HIV in primary CD4+ T cells, without global lymphocyte activation.

### Combination therapy of PC1, kansui, and JQ1 reactivates latent HIV in a primary CD4+ T cell model of HIV latency

The most effective proposed latency reversal strategies include two or more compounds which reactivate latent HIV by different mechanisms [29, 30]. When used in combination, effective concentrations can be reduced to avoid potentially toxic side effects. Because PC1 reactivates HIV through the MAPK pathway, it is ideal to use with a PKC agonist (kansui) and a compound that activates P-TEFb (JQ1).

Latently infected CD4+ T cells were treated with the indicated concentrations of PC1, kansui, and JQ1 either as single, double, or triple combinations. Unstimulated infected cells did not express HSA (Fig 5A–5C, lane 1). Cells were treated with the high concentration of kansui (500 μg/ml) as a positive control, which induced a 15-fold increase in HSA expression (Fig 5A–5C, lane 2). Cells treated with 6 μM PC1 (Fig 5A and 5B, lane 3), kansui (Fig 5A, lane 4), or JQ1 (Fig 5B, lane 4) alone resulted less than 2.5-fold increases in HSA. When 6 μM PC1 was used in combination with kansui or JQ1, HSA expression increased 2-fold over kansui or JQ1 alone (Fig 5A and 5B, lane 5). The additive effect of PC1 and kansui or JQ1 is indicated by the dashed line on each graph, and neither double treatment surpassed this additive effect (Fig 5A and 5B, lane 5) [39]. However, when used as a triple combination, 6 μM PC1 increased expression of HSA 3.5-fold over kansui and 2.5-fold over JQ1 (Fig 5C, lanes 4–6). More importantly, the triple combination of 6 μM PC1, 50 μg/ml kansui, and 0.1 μM JQ1 resulted in a synergistic response, which surpassed the sum of all three single treatments (Fig 5C, lanes 3–6), and was nearly as effective as the high dose of kansui (Fig 5C, lanes 2 and 6). Synergy was confirmed by the Bliss Independence for HIV drug combinations model [29], in which the difference between predicted and observed effects was greater than 1.

**Fig 6:**
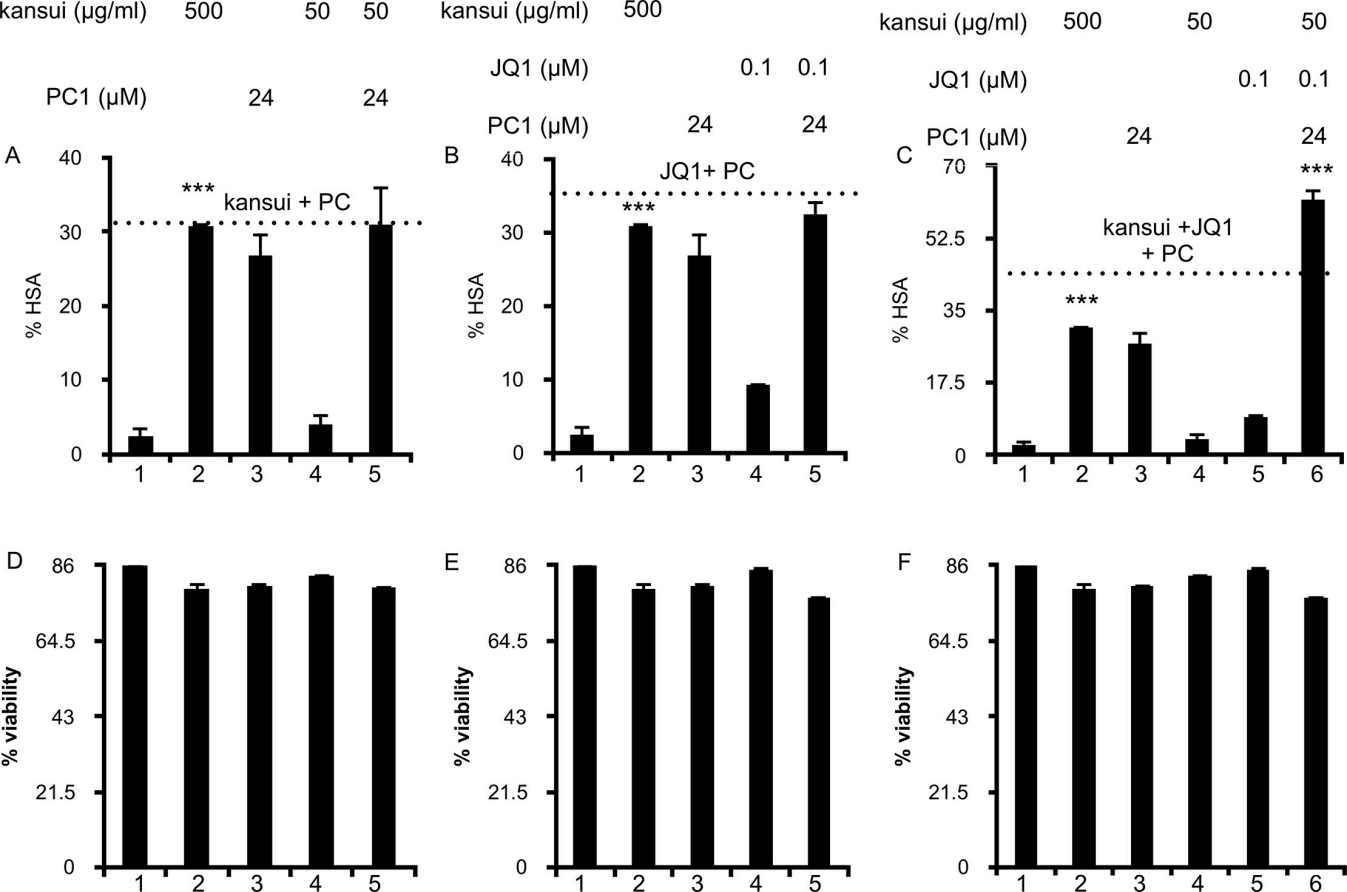
Combination therapy of high dose PC1, kansui, and JQ1 reactivates latent HIV in a primary CD4+ T cell model of HIV latency. Human primary CD4+ T cells were infected with VSV-G-pseudotyped HIV-1 NL4.3 HSA and maintained over 12 days with decreasing concentrations of IL-2 to establish latency as previously described. Uninfected cells were maintained in the same conditions. At 12 days post infection, cells were stimulated for 24 hours with: (A) PC1 (24 μM) and kansui (50 μg/ml) (B) PC1 (24 μM) and JQ1 (0.1 μM) (C) e PC1 (24 μM), kansui (50 μg/ml), and JQ1 (0.1 μM). Cells were stained with anti-HSA antibody and measured by flow cytometry analysis. Data are presented as a fold change in HSA expression over the uninfected control cells. Dashed line is equal to the additive effects (sum % HSA of the single treatments), and a synergistic effect was observed if double and triple treatments surpassed this line. Synergy was validated using Bliss Independence calculation of drug synergy. Experiments were repeated with three independent donors, each with three technical repeats. A representative experiment, from a single donor is presented.(D-F) Uninfected cells were treated as above. Percent viability was estimated using forward/side scatter and the percentage of live lymphocytes in 10,000 total cells was analyzed. Error bars represent standard error of the mean. (*p<0.05, **p<0.01, ***p<0.001).

The triple combination of PC1, kansui, and JQ1 had even greater efficacy with the higher dose of PC1. 24 μM PC1 did not have a synergistic effect in double combinations with kansui and JQ1 (Fig 6A and 6B, lanes 5); the 12.5-fold induction of HSA in each of these treatments was most likely only the effect of PC1 (Fig 6A and 6B, lanes 3 and 5). However, a triple combination with 24 μM PC1 resulted in a 30-fold induction of HSA (Fig 6C, lane 6), and was greater than the additive effects of all three single treatments, as indicated by the dashed line (Fig 6C, lane 6). Synergy was confirmed by the Bliss Independence for HIV drug combinations model [29], in which the difference between predicted and observed effects was greater than 1. Furthermore, the triple combination of 24 μM PC1, 50 μg/ml kansui, and 0.1 μM JQ1 resulted in 2-fold higher induction than the high concentration of kansui alone (Fig 6C, lanes 2 and 6).

Importantly, no loss in cell viability was observed with double or triple combination treatments including 6 μM (Fig 5D–5F) or 24 μM PC1 (Fig 6D–6F). Our previous data indicate that a combination of 50 μg/ml kansui and 0.1 μM JQ1 induces expression of the early T cell activation marker, CD69 without inducing cellular toxicity [21]. PC1 treatment does not induce T cell activation as measured by expression of CD69 (Fig 3A), nor does it activate NF-κB (Fig 2B), which indicates it should not further impact T cell activation by the low dose combination of kansui and JQ1.

Taken together the triple combination of PC1, kansui, and JQ1 effectively reactivated latent HIV by activating different but complimentary signaling pathways. When used as a combination, lower concentrations of all 3 compounds were effective at reactivating latent HIV. However, as PC1 reactivated latent HIV without T cell activation, the higher dose of PC1 may also represent a safer strategy in our triple combination therapy.

## Discussion

In this study we determined that PC1 reactivates latent HIV and works best in a triple combination therapy with kansui and JQ1. PC1 reactivated 2D10 in a dose dependent manner without toxicity. PC1 activated the MAPK signaling pathway, through phosphorylation of ERK. Furthermore, PC1 did not induce CD69 or CD25 expression, indicating that it reactivates HIV without T cell activation. PC1 effectively reactivated HIV in an immortalized cell line and primary CD4+ T cell models of HIV latency. Finally, suboptimal doses of PC1, kansui, and JQ1 reactivated latent HIV more effectively than single doses of any compound, and the highest dose of PC1, when used in triple combination, reactivated latent HIV more effectively than even the highest concentration of kansui.

When considering compounds for therapeutic use, high effective concentrations *in vitro* could translate to even larger clinical doses. In our study, we determined that 18–24 μM PC1 had the highest efficacy, which is a relatively large amount. Clinical dosing strategies could be adjusted to accommodate these effective doses, but this finding is not ideal. However, PC1 treatment does not increase the expression of markers of T cell activation, as measured by CD69 and CD25, and likely would not induce global T cell activation, which indicates that higher therapeutic concentrations should be tolerated without toxic side effects. Additionally, we determined that a triple combinatorial approach, using a lower dose of PC1, reactivated latent HIV as well as a high dose of kansui. Thus, the effective dose of PC1 is significantly reduced when used in combination with kansui and JQ1, at a concentration more ideal for its therapeutic use.

Our study examined three LRAs, which reactivate latent HIV through three different pathways, but when combined reactivate more potently than any single treatment. However, we did not exhaust the complete list of available LRAs to test in combination with PC1. The PKC agonists prostratin and bryostatin activate resting CD4+T cells and latent HIV *in vitro* [40, 41]. However both compounds are too toxic and cost prohibitive to manufacture for widespread clinical applications [42, 43]. Our previous studies indicate that a crude extract of kansui reactivates latent HIV to the same degree as the purified PKC agonist ingenol [21]. Kansui has been used in traditional Chinese medicine for thousands of years with minimal toxicity and is orally bio-available [44]. Other LRAs currently under investigation include: HDACi, such as Panobinostat, Romidepsin, SAHA and valproic acid [45–49]. These all robustly activate HIV in cell line models of latency, by inducing chromatin stress and subsequently releasing of P-TEFb from its inactive complex. All of these compounds reactivate latent HIV through similar mechanisms as JQ1 [17, 18]. Thus, although we did not test an extensive panel of LRAs for our studies, we chose appropriate and representative compounds for an effective combinatorial approach.

Our study utilized immortalized and primary T cell models of HIV latency, but we did not test HIV+ ART suppressed patients. In our previous study we determined that our *in vitro* primary cell latency model provided a good predictor for responses in patient samples [21]. Thus, concentrations of kansui and JQ1 that effectively reactivated latent HIV in this *in vitro* latency model were equally effective in PBMC isolated from HIV+ ART suppressed patients. This *in vitro* model uses gradual reductions in IL-2 concentrations and the removal of T cell receptor engagement over several weeks to bring activated HIV-infected primary CD4+ T cells into a quiescent state. In addition to our model, a number of other primary CD4+ T cells have been designed as a surrogates for studies of proviral latency *in vivo*. A comprehensive review and comparison of several of these models was performed by Spina et al. [19]. In that study, reactivation varied among these models, particularly in the ability to respond to HDACi without additional stimuli, which could indicate that cells in some of these models are not fully resting [18]. In spite of these differences, every model tested reactivated latent HIV following treatment with PKC agonists, albeit at different magnitudes [19]. Our latency model is resistant to reactivation by HDACi and BETi alonge and requires a strong PKC agonist to first increase expression of P-TEFb [21, 22], thus it is appropriate to test the efficacy of the triple combination of PC1, kansui, and JQ1.

Combinatorial therapies have the greatest potential to effectively purge the HIV latent reservoir [29, 30]. PC1, JQ1, and kansui activate cellular transcription factors through distinct pathways, which combined lead to additive effects on HIV reactivation as well as to reduced effective concentrations of individual compounds. By combining different compounds, lower doses of a kansui can be administered, reducing global T cell activation. Furthermore, treatment with JQ1 activates P-TEFb transiently, which induces a feedback loop that is characterized by increased expression of Hexim1 and the subsequent inactivation of P-TEFb in the 7SK snRNP [50]. In the meantime, Tat is synthesized, which can utilize P-TEFb even from the inactive complex [10]. Whereas HIV transcription is maintained, cells return to their resting state. Additionally, PC1 activates through MAPK signaling without T cell activation. Therefore together this combination may prevent inflammatory gene expression and the resulting cytokine storm, while still potently reactivating latent HIV. Double combinations of LRAs have been extensively researched, including combinations of PKC agonists and HDACi/BETi, which resulted in synergistic reactivation [23, 29, 30, 33]. Our triple combinatorial approach expands on this research and includes compounds which reactivate latent HIV at higher concentrations. When used together at lower concentrations, all three potently reactivate latent HIV at more manageable therapeutic concentrations.

Kansui and PC1 are both compounds derived from natural plant sources, which represent affordable, readily available raw materials which can easily be made into therapeutic agents [51]. Kansui has been used for thousands of years in traditional Chinese medicine for treatment of fluid retention, ascites, and cancer; consumed as a tea in its raw form [52–54]. Kansui is a member of the Euphorbia family of plants, which are found in abundance world–wide. Euphorbia, are the main plant source of ingenol, which in addition to its ability to reactivate latent HIV, has been developed into a topical treatment of actinic keratosis world-wide and used as the cancer therapeutic Aveloz in Brazil [53, 55]. PC1 is found in a variety of plant sources including grape, apple, cinnamon, and cacao, which are all ubiquitous sources of raw material [56]. The plant sources of PC1 have natural metabolic and anti-inflammatory properties as well as reported cardiovascular and anti-cancer benefits [57–60]. While natural product based therapeutics are often viewed as alternative medical treatments, scientific discovery of therapeutic compounds has a rich history of plant derived drugs, including aspririn, taxol, and quinine [51]. Taking advantage of these natural products has the potential to yield affordable and more easily scalable production of LRAs.
